# Multi-branch deep learning neural network prediction model for the development of angular biosensors based on sEMG

**DOI:** 10.3389/fbioe.2024.1492232

**Published:** 2024-10-11

**Authors:** Liman Yang, Zhijun Shi, Ruming Jia, Jiange Kou, Minghua Du, Chunrong Bian, Juncheng Wang

**Affiliations:** ^1^ School of Automation Science and Electrical Engineering, Beihang University, Beijing, China; ^2^ Institute of Stomatology, First Medical Center, Chinese PLA General Hospital, Beijing, China; ^3^ Department of Oncology, Caoxian People’s Hospital, Heze, China

**Keywords:** lower extremity exoskeleton, surface electromyography, gait recognition, joint angle prediction, neural network

## Abstract

**Introduction:**

Human gait motion intention recognition is very important for the lower extremity exoskeleton robot to accurately synchronize and respond to the user’s natural motion. And motion intention recognition is generally performed through sEMG. Deep learning neural networks perform well in dealing with high-dimensional data and nonlinear relationships such as sEMG, but different deep learning neural networks have their own advantages in dealing with different types of data. Therefore, a multi-branch deep learning neural network, which enables different neural networks to process different feature items, could achieve more accurate and efficient motion intention recognition. The purpose of this study is to 1) Establish a multi-branch deep learning neural network model to achieve accurate gait recognition and effective estimation of joint angles. 2) Quantify the performance of the multi-branch deep learning neural network model in gait recognition and joint angle prediction using sEMG.

**Methodology:**

This study involved the collection of sEMG and plantar pressure data during walking in human subjects. Firstly, the collected signals are filtered and denoised to ensure the quality and reliability of the data. Calculate the time domain features and the frequency domain features to capture the key information of gait. Then, using the sensitivity difference of different structural neural networks to different feature data, a multi-branch deep learning neural network model is developed, in which the extracted features are used as the input of the model. The output of the model includes gait cycle and joint angle, so as to realize the accurate recognition of human gait and the effective estimation of joint angle.

**Results:**

The results show that the proposed method has high accuracy in identifying human gait and estimating joint angles. The multi-branch neural network model successfully integrates time-domain and frequency-domain features and provides reliable prediction of gait cycle and joint angle. The highest accuracy of gait recognition is 95.42%, the lowest is 90.11%, and the average is 92.16%. The average error of joint angle estimation is 3.19.

**Discussion:**

This study designed a human walking gait recognition and joint angle prediction model to achieve accurate human lower limb motion intention recognition.The model can be integrated into the sEMG sensor to design a angular biosensors, which can predict the human joint angle in real time.

## 1 Introduction

The current global population aging is becoming more and more serious, resulting in an increase in age-related diseases and injuries, such as osteoarthritis, neurodegenerative diseases (such as Parkinson‘s disease), stroke sequelae, etc. These conditions will lead to inconvenience of limb movement and affect the daily living ability and independence of the elderly, followed by more and more lower limb disorder groups (Billot et al., 2020). Because the rehabilitation robot can provide efficient rehabilitation training, meet the rehabilitation training needs of most hemiplegic patients, and alleviate the shortage of rehabilitation resources (Bertani et al., 2017), it has attracted more and more attention. Lower limb rehabilitation robots can be roughly divided into two types: end-driven and exoskeleton (Díaz et al., 2011). The end-driven type has low reproduction accuracy and is difficult to adapt to the gait trajectory of different populations. The exoskeleton has a large number of degrees of freedom, which can flexibly and accurately reproduce the gait trajectory of the lower limbs. Therefore, it has become a research hotspot in the field of robotics. Exoskeleton-type rehabilitation robots require human-computer interaction and collaboration (Meng et al., 2015). With the development of biological information technology, biological signals such as electroencephalogram and electromyography have been widely used in the interface development of human-computer systems (Gopura et al., 2016). Since the surface EMG signal is directly related to the contraction state of the muscle, it can be used to monitor muscle movement in real time and identify gait (Agostini et al., 2020). At the same time, compared with other signals, surface electromyography signals are generated before the actual movement of the human body. Therefore, human gait recognition based on surface electromyography signals has higher real-time performance and portability (Kyeong et al., 2022). Engineers use surface electromyography to develop a feasible human-machine system interface.

In recent years, surface electromyography (sEMG) signals have been widely used to extract human motion information. There are two main methods. For the first method, researchers used sEMG signals to identify different motion patterns of human limbs. Therefore, higher recognition rate and more motion patterns are two goals, and feature extraction methods and classification algorithms are the focus of research (McCool et al., 2015; Mesa et al., 2014; Lloyd and Besier, 2003; Zhang et al., 2012; Ardestani et al., 2014). However, only a limited number of motion modes can be identified from the sEMG signal, and the recognition result is only used as the switching signal of the robot. This can only enable the rehabilitation robot to perform trigger training (Chen et al., 2023). Although it can meet the training requirements, to a certain extent, the safety and comfort of this control cannot be guaranteed. In addition, the traditional trigger control may only ensure that patients can learn the trigger behavior, but not the whole rehabilitation action (Maciejasz et al., 2014). The purpose of rehabilitation training is not only to maximize the number of repetitions, but also to maximize the concentration of patients' attention and efforts (Marchal-Crespo and Reinkensmeyer, 2009). In contrast, the second method is to use sEMG signals to continue to estimate motion variables, which can achieve smooth motion control. Many methods have been proposed to establish the relationship between sEMG signals and motion variables. For example, the forward biomechanical model is constructed and calibrated, and the joint torque is calculated using sEMG (Aung and Al-Jumaily, 2012). Artificial neural networks (Rogers et al., 2011; Tsai et al., 2014; Hermens et al., 2000) and polynomial fitting (Han et al., 2024) are also used to map sEMG signals to joint angles or joint torques. This can achieve a more feasible human motion control strategy (Chen et al., 2018) -continuous control.

At present, the sEMG signal method based on continuous motion estimation mainly focuses on physiological muscle model and neural network model. Systems based on physiological models include kinematic models (Borbély and Szolgay, 2017), kinetic models (Clancy et al., 2011), and musculoskeletal models (Buongiorno et al., 2018). HILL muscle model is the most widely used. Although the above model has a good fitting effect on the regression of single joint motion, it is difficult to train the model due to a large number of physiological parameters that are difficult to measure. In addition, when modeling multiple joints with multiple degrees of freedom, the redundant control of human muscles makes the model very complex (Bi and Guan, 2019).

Compared with the physiological muscle model, neural network model is a more direct and convenient method. The strong fitting ability of neural network makes continuous control based on electromyography possible (Ma et al., 2024). Zhang Feng and other researchers used BP neural network to predict the continuous motion angle of human lower limb joints using sEMG. The researchers tested the method under different motion speeds and load conditions to test the effect of the method on predicting joint angles. Based on the neuromusculoskeletal model of Hill muscle force model, Sartori M et al. (Sartori et al., 2017) proposed a generalized model that integrates multiple muscles-multiple musculoskeletal units-multiple degrees of freedom. The researchers recorded the sEMG signal from the thigh muscle group, established the dynamic model of the multi-musculoskeletal unit, and used the virtual annealing algorithm to calibrate the parameters. Finally, the muscle force output by the model can be used to predict the multi-degree-of-freedom joint torque. David et al. (Quintero et al., 2018) proposed a method for predicting continuous joint angles using sEMG to control electric knee and ankle prostheses. Zhu M and Guan X et al. (Zhu et al., 2023) proposed a new model combining CNN and LSTM to predict the knee joint angle, combining feature extraction and time series regression for deep learning, making full use of the spatial and temporal correlation of sEMG signals to make the prediction model more accurate and effective. Such predictive signal methods can provide more accurate control for lower limb exoskeleton robots.

Deep learning methods such as convolutional neural network (CNN) and recurrent neural network (RNN) have excellent performance in dealing with high-dimensional data and nonlinear relationships such as sEMG signals. They can automatically extract features and reduce the difficulty of feature extraction (Hu et al., 2018). However, different types of deep learning neural networks have their own advantages in dealing with different types of data and task scenarios. It is difficult to achieve satisfactory recognition results with only one neural network architecture (Alzubaidi, 2021).

Therefore, this paper designs a multi-branch deep learning neural network model, which takes the characteristics of sEMG signals as input, gait and joint angle as output, and uses different neural network architectures to process different feature items, giving full play to the advantages of each neural network, so as to improve the accuracy of gait recognition and joint angle estimation. This technology can help to design more accurate angular biosensors, which can be applied to lower limb rehabilitation robots to provide more accurate continuous control instructions for lower limb rehabilitation robots. By collecting the sEMG signal of the patient in real time and predicting the joint angle, the rehabilitation robot can adjust the training intensity and mode according to the specific situation of the patient to achieve a more personalized rehabilitation treatment plan. It can even analyze the functional status of muscles through sEMG signals to help doctors assess the degree of muscle damage and rehabilitation process of patients.

The rest of this article is organized as follows. The multi-branch deep learning neural network is constructed in Section 2. The effect of the model on gait recognition and joint angle prediction is evaluated in Section 3. Finally, Section 4 concludes this article.

## 2 Methods

### 2.1 Data collection

**FIGURE 1:**
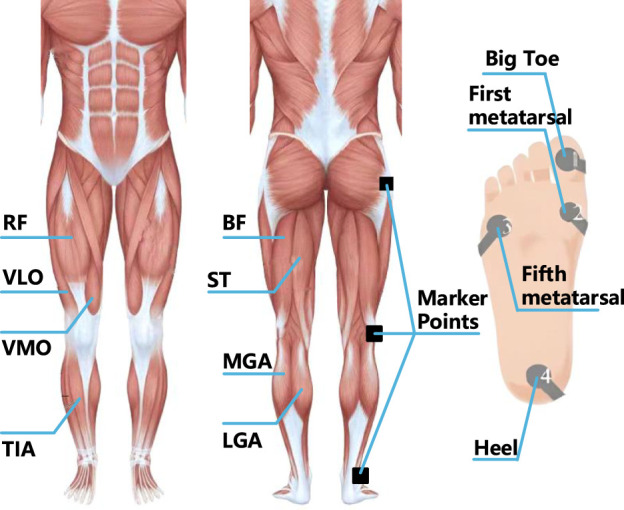
Position of signal acquisition sensor.

This study analyzes the functions of various muscles in different lower limb movements. According to relevant experiments and previous studies of other researchers, eight muscles of the right lower limb that are highly active in the gait cycle are selected as signal acquisition positions to facilitate access to useful information (Trinler et al., 2018). These muscle groups and muscles include the quadriceps femoris, the posterior thigh muscle group, the gastrocnemius muscle and the anterior tibial muscle. In the analysis of lower limb movement, it is very important to obtain gait information to determine the current stage of movement, and the plantar force signal can reflect the gait cycle (Yang et al., 2020). Therefore, this study completes the collection of plantar pressure signals by placing the plantar pressure switch on the heel, fifth metatarsal, first metatarsal and big toe of the left and right soles. In the experiment, volunteers walked on a treadmill at three different speeds of 3, 3.5 and 4 km/h, each speed repeated three times for 6 min. The Noraxon Desktop DTS-8 wireless sensor system is used to collect sEMG signals and plantar pressure signals at a sampling frequency of 1.5 KHz. In addition, the optical motion capture device is used to record the trajectory of 16 marker points of the lower limbs of the human body, and then the angles of the hip joint, knee joint and ankle joint of the lower limbs are obtained. The position of the signal acquisition sensor is shown in Figure 1.

### 2.2 Data processing

**FIGURE 2:**
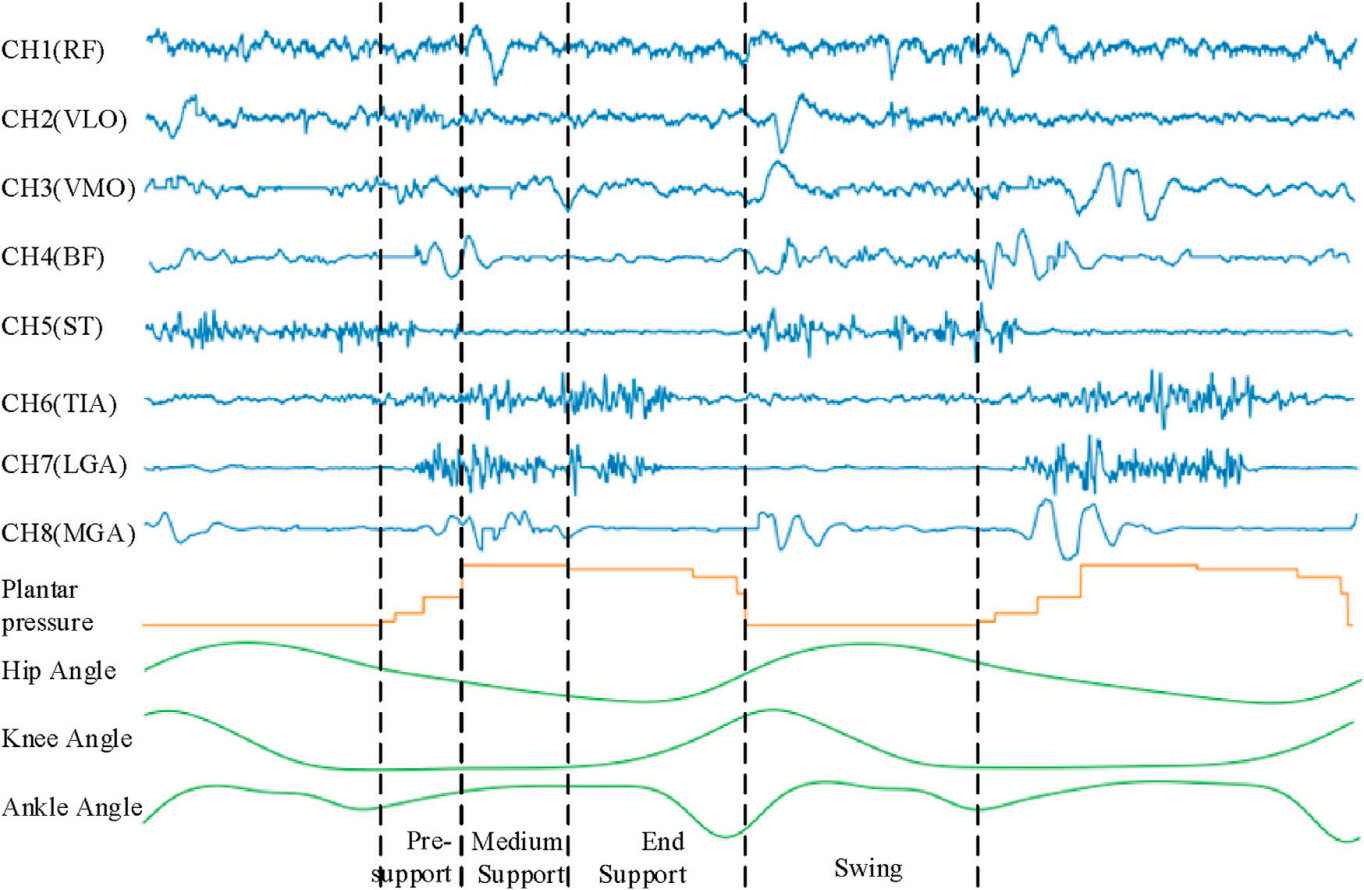
The relationship between sEMG signal, plantar pressure signal, joint angle signal and gait.

The sEMG, plantar pressure and joint angle signals obtained by related sensors, pressure switches and motion capture devices are sent to the desktop receiver and stored as raw data. The collected original signal is shown in Figure 2. The sEMG of the human thigh muscle is channel 1 to channel 5 (CH1-CH5), which mainly plays a role in controlling the movement of the hip joint and knee joint, while the calf muscle of the human body is mainly related to the movement of the ankle joint. The human gait is divided into four walking gaits: swing period (Swing, SW), initial contact period (Initial Contact, IC), middle support period (Mid Stance, MSt) and terminal support period (Terminal Stance, TSt). In different gaits during a walking cycle, sEMG at different positions show amplitude changes. The sEMG of the thigh-related muscles undergo more obvious amplitude changes in the early stages of SW and IC, which is due to the concentration of strength of the thigh muscles in the movement of the hip and knee. On the contrary, the sEMG signal of the calf-related muscles has a relatively high frequency change during IC, MSt and TSt which is due to the need of the calf to provide support and ankle rotation torque. In a walking cycle, the angular range of motion of the hip is −15°∼30° , the knee is −12°∼45° , and the ankle is −20°∼8° .

During the signal acquisition process, it is highly susceptible to interference from external environmental factors, equipment power supply variations, and physiological artifacts. Therefore, it is necessary to further filter and denoise the collected signals to obtain higher quality and more reliable signals. In this study, the second-order Butterworth filter is selected. On the one hand, it has smooth frequency response characteristics, which can filter the signal smoothly (Shouran, 2020). While removing noise, it can maintain the stability and continuity of the signal and reduce the loss of information as much as possible. On the other hand, it can flexibly adjust the passband and stopband width of the filter to meet the filtering requirements of different frequencies, and it also has certain advantages in real-time processing and large-scale data processing.

**FIGURE 3:**
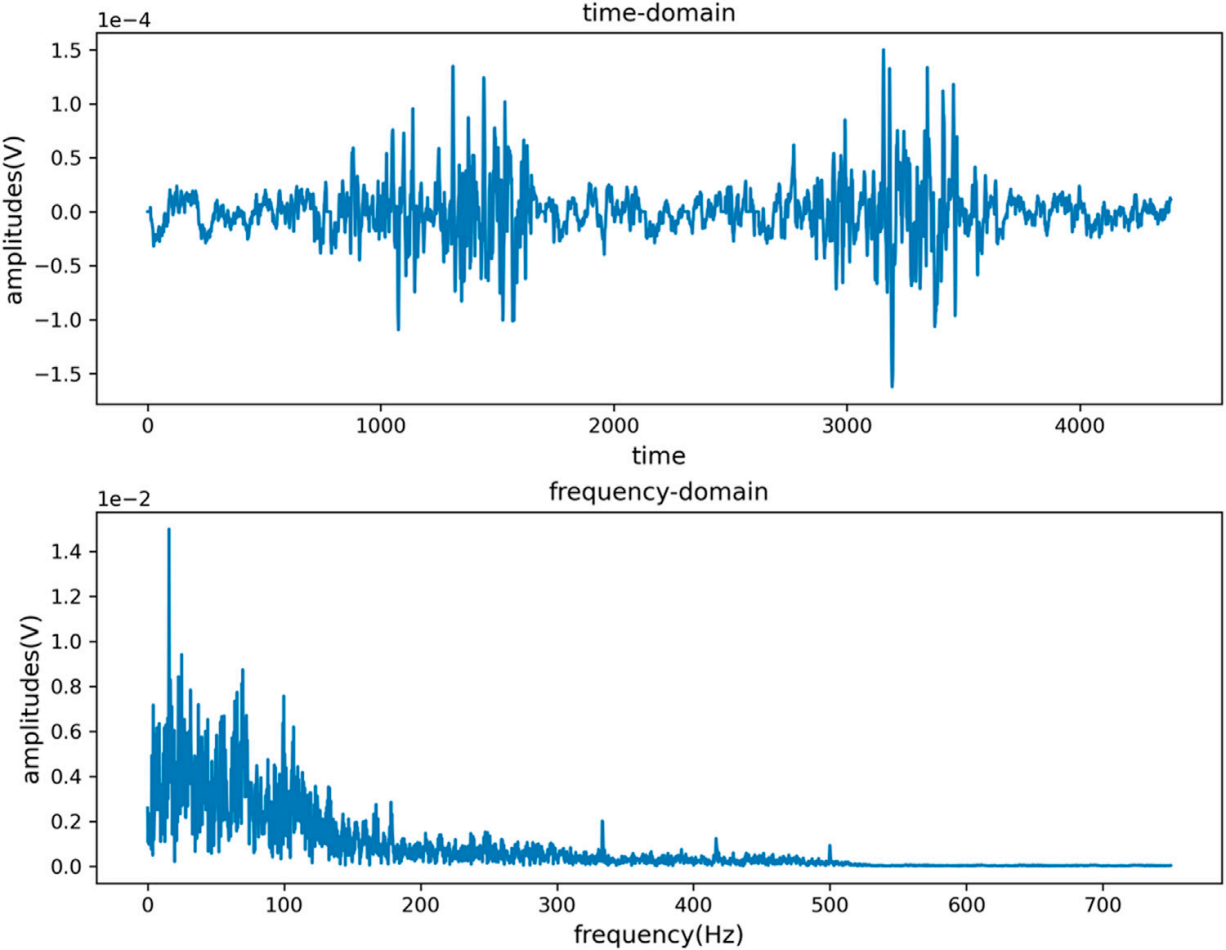
Time domain and frequency domain diagram of TIA channel original signal.

**FIGURE 4:**
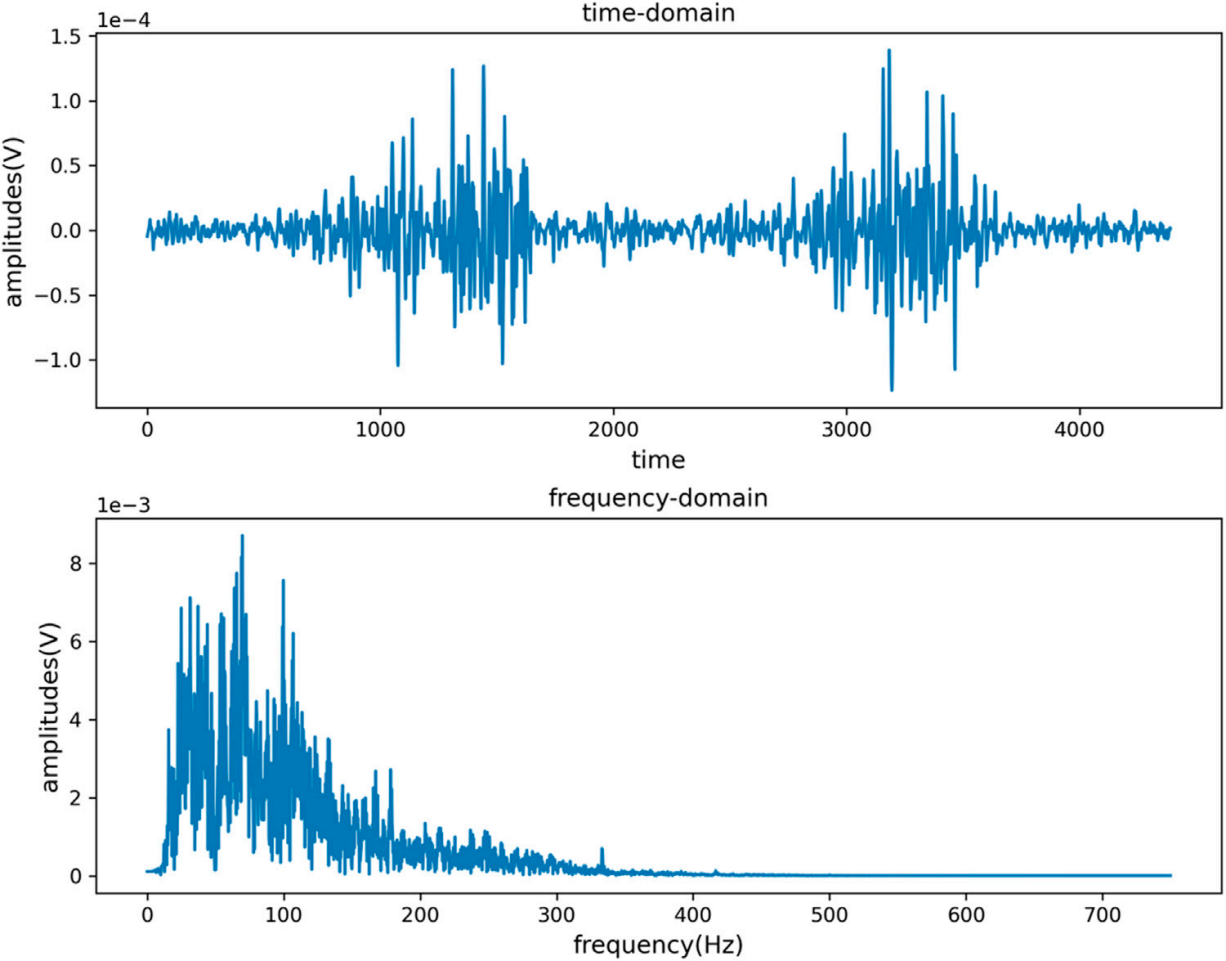
Time domain and frequency domain diagram of signal after TIA channel filtering and noise reduction.

Since the main energy frequency range of the sEMG signal is between 0–500 Hz, a 20–300 Hz band-pass filter and a 49–51 Hz band-stop filter are respectively set to eliminate the interference caused by low frequency, high frequency and power frequency signals. Figures 3, 4 are the time domain and frequency domain diagrams of the original and filtered noise reduction of the sEMG signal TIA channel, respectively. The figures indicate that the signal after filtering and noise reduction is smoother, and the 50 Hz power frequency interference is basically eliminated.

**FIGURE 5:**
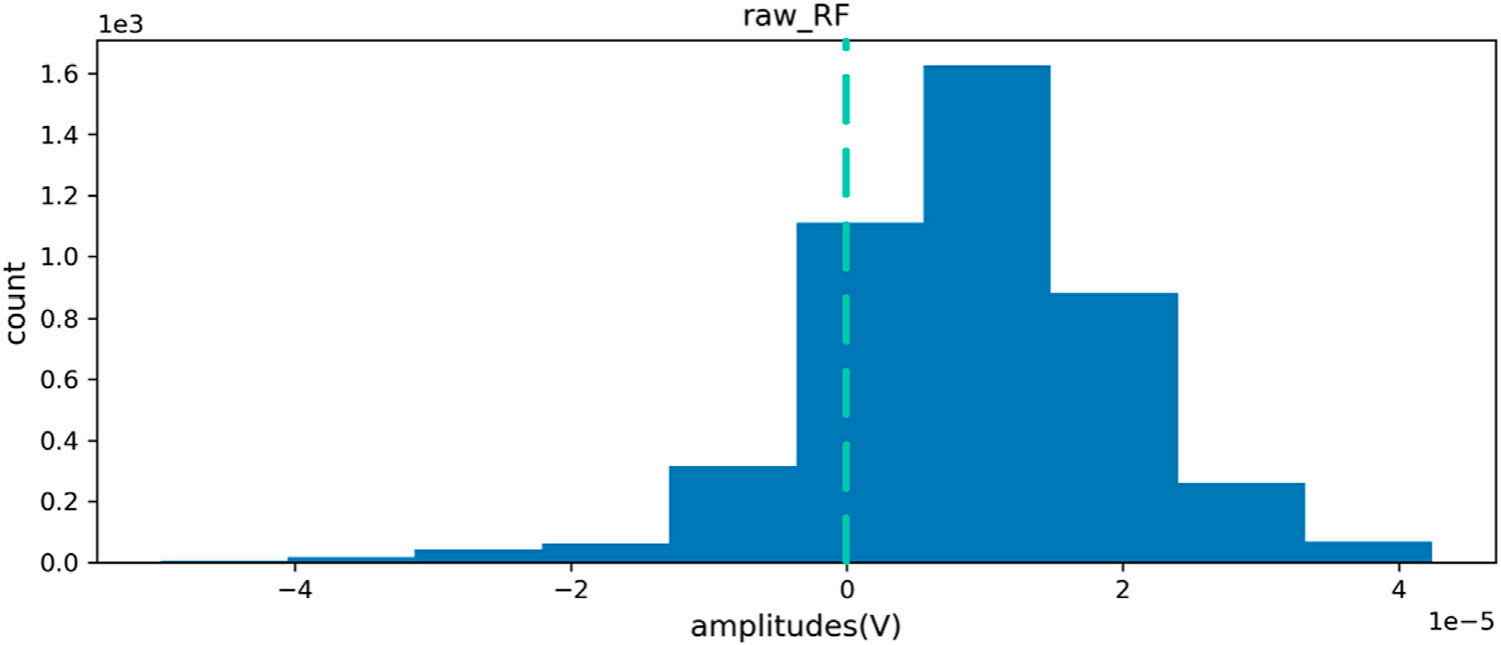
RF channel data distribution before standardization.

**FIGURE 6:**
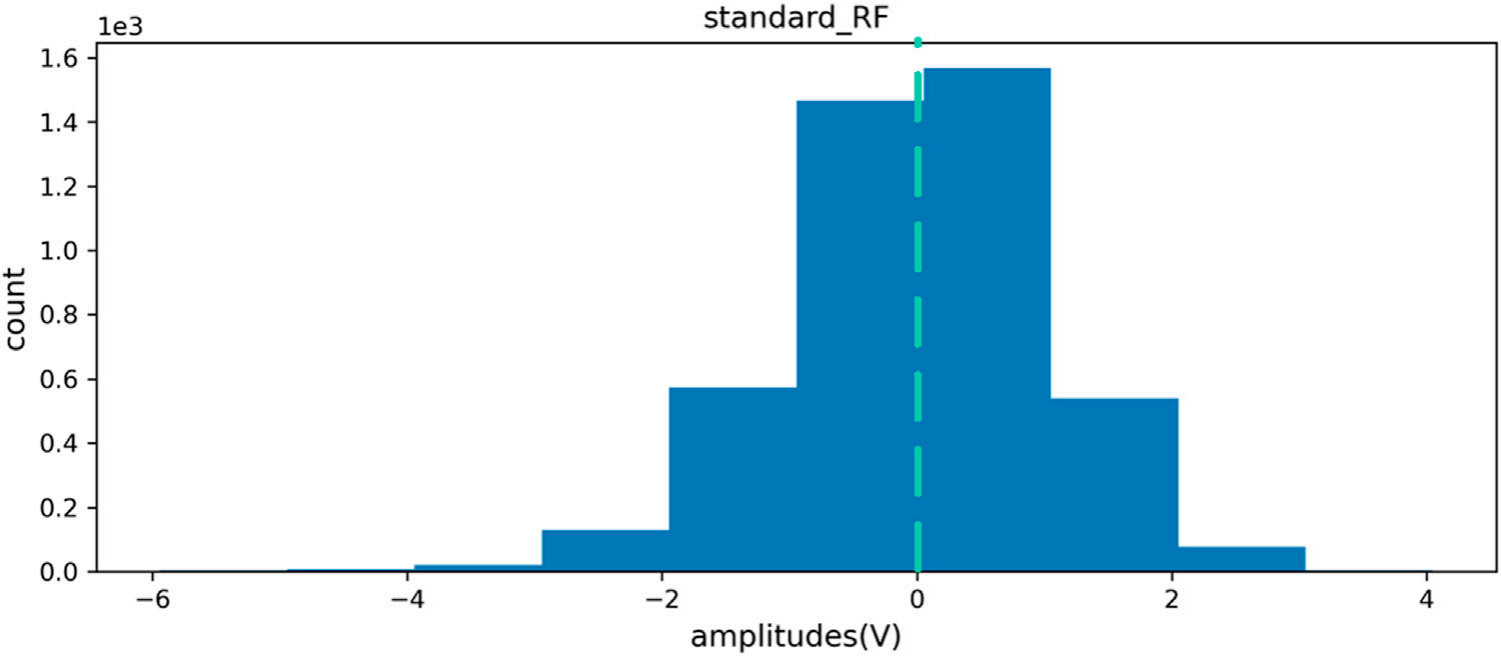
RF channel data distribution after standardization.

After filtering and noise reduction, the zero standardization method is used to standardize the signal data. While eliminating the individual differences between different measured objects and the deviations generated during the acquisition process, the distribution information of the original data can be better retained. Figures 5, 6 are the distribution maps of RF channels of sEMG before and after standardization. The figures indicate the standardized data distribution is symmetrical. The data distribution on the left and right sides with the mean as the center is similar and close to the normal distribution, which is helpful for the analysis of subsequent data characteristics and the training of subsequent human gait recognition and joint angle estimation algorithm models.

**FIGURE 7:**
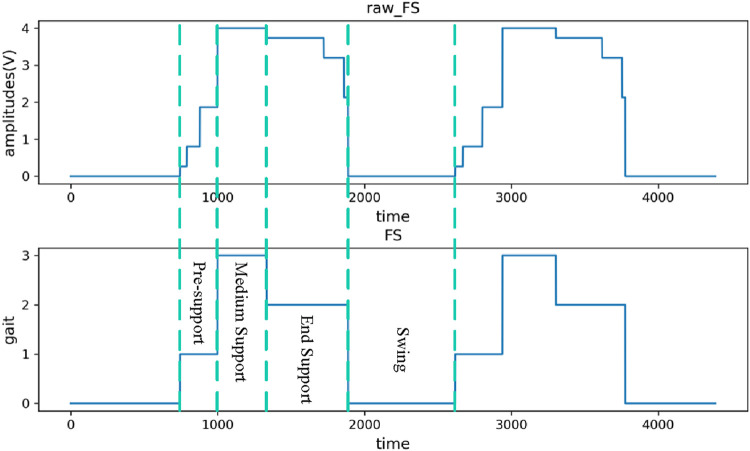
Plantar pressure signal converted to four gaits.

For the plantar pressure signal, this study uses smooth filtering technology to denoise it, and then uses the threshold determination method to extract the gait cycle, and converts the plantar pressure signal into four walking gaits as the label data for subsequent model training. Figure 7 is the signal diagram before and after the plantar pressure signal is converted into four gaits.

### 2.3 Multi-branch deep learning neural network

#### 2.3.1 Feature extraction

The sEMG signal after the above processing still contains a large amount of data information, and directly input it into the model will reduce the training speed and recognition accuracy. Therefore, before the model training and recognition, the signal is usually extracted for the purpose of obtaining effective feature information and improving the model performance.

For sEMG, several typical characteristics such as time domain, frequency domain and time-frequency domain can be obtained in a given time window. Table 1 shows several commonly used sEMG signal features in human gait recognition selected in this study (Qin and Shi, 2020). The extracted features can be combined into vectors directly as the input of the gait recognition model, and can also be fused with other types of features before entering the model.

**TABLE 1:** Characteristics of sEMG.

Feature name	Feature type	The information reflected
wave length	time domain	Complexity and waveform changes
Variance	time domain	Energy distribution and fluctuation degree
average absolute value	time domain	Total muscle activity intensity
root mean square	time domain	Muscle activity intensity
Number of zero crossings	time domain	Frequency change and volatility
short time fourier transform	time and frequency domain characteristics	local frequency information

#### 2.3.2 Time domain feature extraction

Wave length (WL):

$$\text{WL}=\sum_{n=2}^{N}\left|y\left(n\right)-y\left(n-1\right)\right|$$ (1)

Variance (VAR):

$$VAR=\frac{1}{N-1}\sum_{n=1}^{N}{\left(y\left(n\right)-\bar{y}\right)}^{2}VAR=\frac{1}{N-1}\sum_{n=1}^{N}{\left(y\left(n\right)-\bar{y}\right)}^{2}$$ (2)

Average absolute value (MAV):

$$MAV=\frac{1}{N}\sum_{n=1}^{N}\left|y\left(n\right)\right|$$ (3)

Root mean square (RMS):

$$\text{RMS}=\sqrt{\frac{1}{N}\sum_{n=1}^{N}y^{2}\left(n\right)}$$ (4)

Number of zero crossings (ZC):

$$\begin{array}{rr} & \text{ZC}=\sum_{n=2}^{N}\zeta \left(n\right)\\ & \zeta \left(n\right)=\left\{\begin{array}{l} 1,\text{ify}\left(n\right)\times y\left(n-1\right)<0\text{and}\left|y\left(n\right)-y\left(n-1\right)\right|\geq \varepsilon \\ 0,\text{else} \end{array}\right. \end{array}$$ (5)

Where, $y\left(n\right)$ is a certain point in time signal value. Finally, the time-domain characteristics of sEMG can be represented by a 5 × 8 matrix.

**FIGURE 8:**
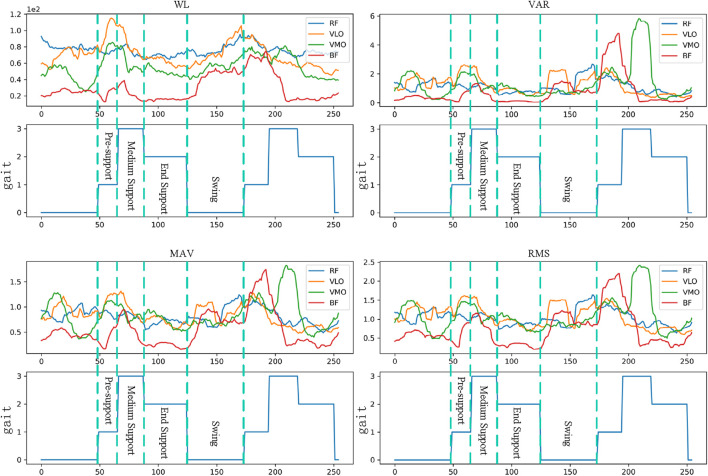
Changes of time domain features under continuous gait.

The time-domain characteristic changes of sEMG based on Equations 1–5 in continuous gait are described in Figure 8. Regarding waveform length, the sEMG signal of the biceps femoris exhibits higher amplitude during the early stages of the swing and support phases, whereas the sEMG signal of the medial trapezius shows greater amplitude during the mid-support phase. Concerning the average absolute value characteristics, the sEMG signal of the medial trapezius demonstrates significant amplitude in the mid-support phase. Conversely, the sEMG signal of the biceps femoris shows substantial amplitude before the support phase and during the transition from late support to the swing phase. The variations in variance and root mean square signal characteristics follow a similar pattern.

However, the time-domain features are weak in terms of frequency information and time-varying, which leads to the inability to fully capture the characteristics of the signal in some complex scenarios. Therefore, after extracting the time-domain features of sEMG, this study further extracts its time-frequency domain features.

$$\left[\begin{array}{cccccccc} WL_{RF} & WL_{VLO} & WL_{VMO} & WL_{BF} & WL_{ST} & WL_{TIA} & WL_{LGA} & WL_{MGA}\\ MAV_{RF} & MAV_{VLO} & MAV_{VMO} & MAV_{BF} & MAV_{ST} & MAV_{TIA} & MAV_{LGA} & MAV_{MGA}\\ VAR_{RF} & VAR_{VLO} & VAR_{VMO} & VAR_{BF} & VAR_{ST} & VAR_{TIA} & VAR_{LGA} & VAR_{MGA}\\ RMS_{RF} & RMS_{VLO} & RMS_{VMO} & RMS_{BF} & RMS_{ST} & RMS_{TIA} & RMS_{LGA} & RMS_{MGA}\\ ZC_{RF} & ZC_{VLO} & ZC_{VMO} & ZC_{BF} & ZC_{ST} & ZC_{TIA} & ZC_{LGA} & ZC_{MGA} \end{array}\right]$$

#### 2.3.3 Time-frequency domain feature extraction

In this study, Short-Tim Fourier Transform (STFT) is selected to analyze the time-frequency characteristics of sEMG. The main idea is to perform Fourier transform on each time segment of the signal, so as to obtain the frequency components of each time segment and understand the frequency characteristics of the signal at different time points. Its calculation formula is Equation 6:

$$\text{STFT}\left\{x\left(n\right)\right\}\left(m,\omega \right)\equiv X\left(m,\omega \right)=\sum_{n=-\infty }^{\infty }x\left[n\right]\omega \left[n-m\right]e^{-j\omega n}$$ (6)

**FIGURE 9:**
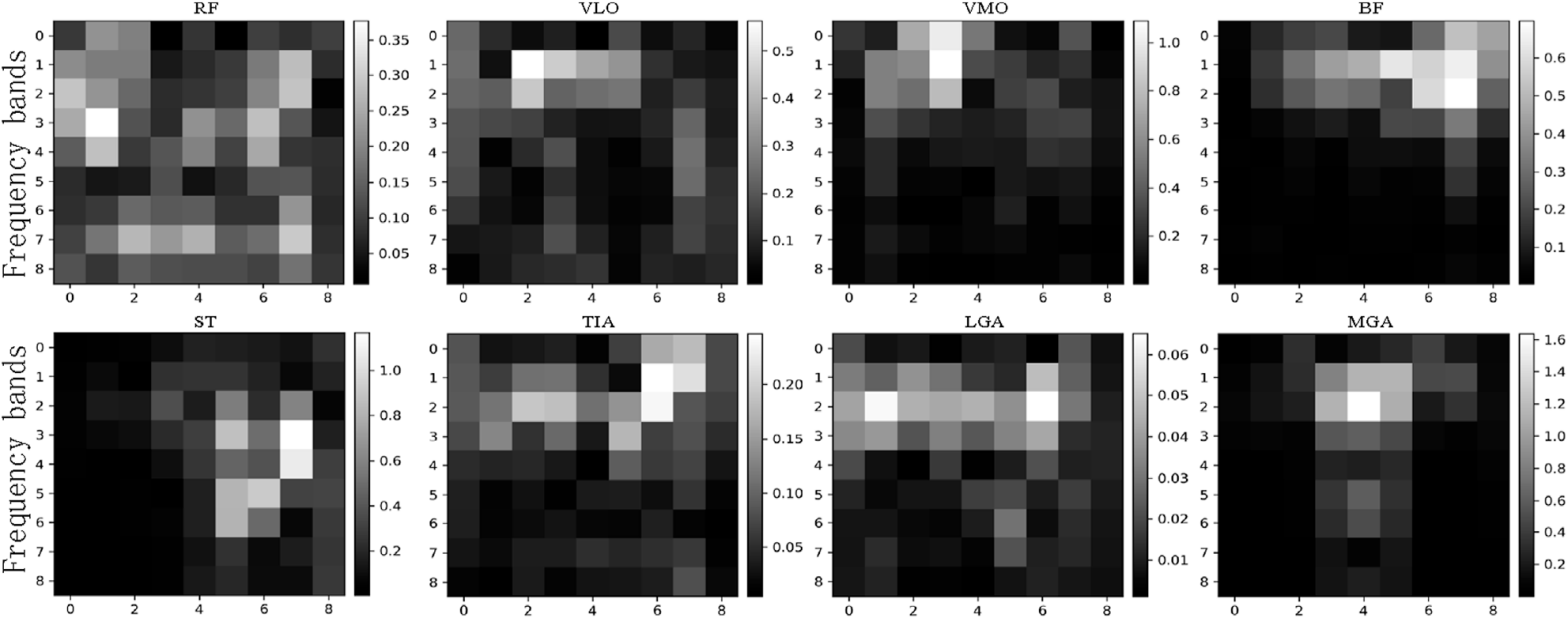
Spectrum of single segment signal.

In this study, the Hann window and the 32-point overlapping 64-point STFT method are used to process the data. The spectrum of each segment contains 33 different frequency bands (0–750.00 Hz) and 6 time periods. Therefore, the spectrum of each segment can be expressed as a matrix of 33 × 9 × 8 (frequency × time × channel). Since the energy of most surface muscle electrical signals is mainly concentrated in the range of 0–200Hz, only the first 9 lines (0–187.50 Hz) of the spectrogram need to be retained. Therefore, the dimension of each spectral graph matrix is 9 × 9 × 8 (frequency × time channel). The spectrum of the 8-channel surface EMG signal processed by the STFT method is shown in Figure 9. These processed spectrograms, together with the aforementioned extracted time-domain features, will be used as a training data set for the neural network to help the network learn to identify the characteristics of different gait cycles and predict the corresponding joint angles, and some will be used to test the recognition performance of the neural network model.

#### 2.3.4 The overall architecture of multi-branch deep learning neural network

**FIGURE 10:**
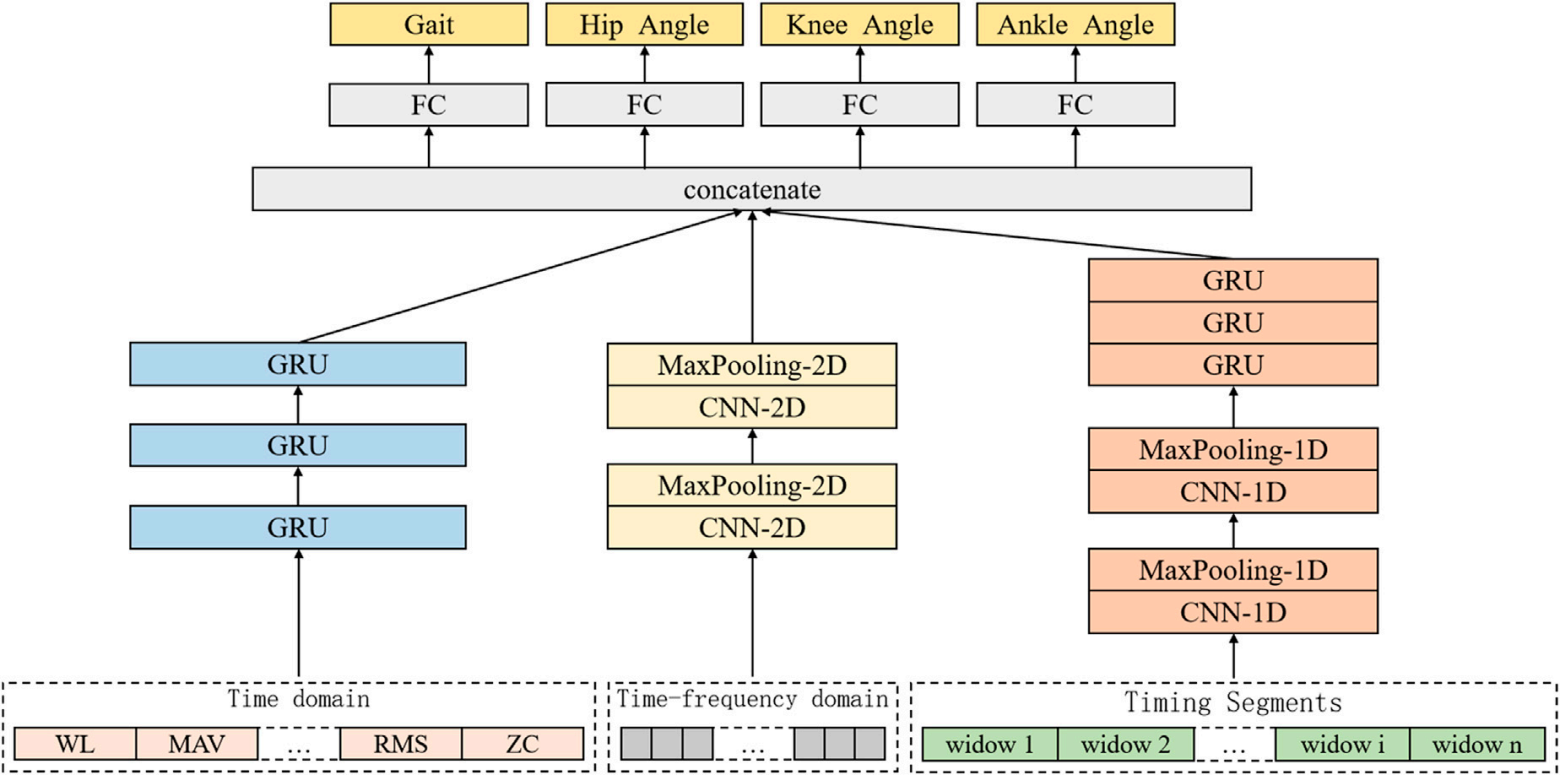
Architecture of human motion intention recognition system based on multi-branch deep learning neural network.

Multi-branch deep learning neural network is a complex and efficient network structure, which can process and analyze input data at multiple levels. Based on the extraction of time domain and time-frequency domain features of sEMG, this study designs a multi-branch deep learning neural network including three branches for feature extraction and learning of different types of data, so as to realize the recognition and prediction of lower limb walking gait and joint angle. The input of the whole network is the sEMG signal after preprocessing and feature extraction, which is the time domain feature, time-frequency feature and time sequence fragment of the sEMG signal, respectively. These three feature items are input into three branch neural networks respectively. After the fusion layer, it is merged into a vector and input into the corresponding fully connected network (FC) respectively. Finally, the human gait recognition result and the estimated angle of hip joint, knee joint and ankle joint are obtained. Among them, the branch for gait recognition needs to go through a layer of SoftMax activation function, and finally calculate the probability of recognized walking gait. The overall design of its architecture is shown in Figure 10.

Among them, the first branch designs a one-dimensional CNN architecture for feature learning of sEMG with timing and high-dimensional characteristics, and its input is the timing segment of sEMG. After a series of convolution layers, activation functions and pooling layers, the one-dimensional CNN can learn the relevant timing features in the sEMG signal timing segment. Then these features are input into the three-layer gated recurrent unit (GRU) RNN for further time series feature learning. The second branch aims at the time-frequency domain feature of sEMG signal spectrum, and designs a two-dimensional CNN architecture to learn its features. The input is the spectrum extracted from the sEMG signal sequence segment. After a series of convolutional layers, activation functions and pooling layers, the two-dimensional CNN can learn the relevant time-frequency domain features in the sEMG signal spectrum, providing richer information for the identification and estimation of joint angles.

#### 2.3.5 One-dimensional CNN architecture design

One-dimensional CNN is commonly used to process time series data. It has translation invariance. It can perform convolution operations through local receptive fields, automatically learn and extract local features in the signal, so as to effectively capture the time series characteristics of sEMG. Therefore, for the filtered and normalized sEMG time series fragments, in this study, a one-dimensional CNN architecture is designed, which is composed of two layers of one-dimensional CNN, supplemented by one-dimensional pooling layer for efficient feature learning and selection.

Corresponding to the 8-channel timing fragment, its convolution layer can be expressed as Equation 7:

$$y_{i,l}=\sigma \left[\sum_{n=1}^{N}\sum_{j=i}^{i+2}w_{j,n,l}u_{j,n}+b_{l}\right]$$ (7)

Where the input is defined as $\left\{U_{i}=\left[u_{i,1}u_{i,2}...u_{i,N}\right]\left|i=1,2,...,I;N=1,2,...,8\right.\right\}$ , and the convolution kernel is defined as $\left\{W_{l}=\left[w_{i,1}w_{i,2}...w_{i,N}\right]\left|i=1,2,...,I;N=1,2,...,L\right.\right\}$ . N is the number of channels, i is the length of the time series fragment, and L is the number of convolution kernels. A convolution kernel with a size of 3 and a step size of 1 is selected, and $b_{l}$ is bias, σ is a nonlinear activation function.

**FIGURE 11:**
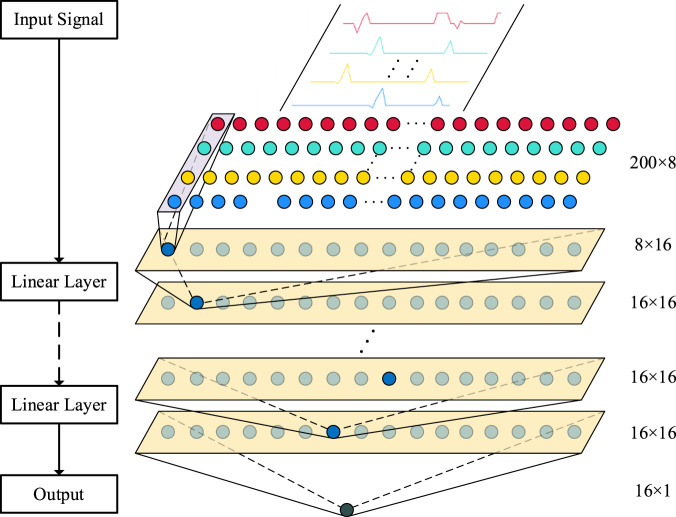
One-dimensional CNN architecture.

A one-dimensional pooling layer is added after each one-dimensional convolution layer to reduce the data dimension and reduce the amount of calculation. Figure 11 is the one-dimensional CNN structure designed in this study.

#### 2.3.6 Two-dimensional CNN architecture design

Two-dimensional CNNs are often used to process images or data with two-dimensional structures, which can effectively capture the local features and patterns of sEMG spectrograms. Therefore, for the spectrum obtained by extracting time-frequency domain features from sEMG, this study designs a two-dimensional CNN architecture for further feature learning.

The input of the two-dimensional CNN architecture corresponds to the 8-channel spectrogram. The input is defined as $X_{I,J,C}$ , the convolution kernel is $W_{I,J,C,L}$ , I is the height, J is the width, C is the channel, and L is the number of convolution kernels. The convolution kernel with a size of 3 × 3 and a step size of 2 is selected, and its convolution layer can be expressed as Equation 8:

$$y_{l}=\sigma \left(\sum w_{i,j,c,l}x_{i,j,c}+b_{l}\right)$$ (8)

**FIGURE 12:**
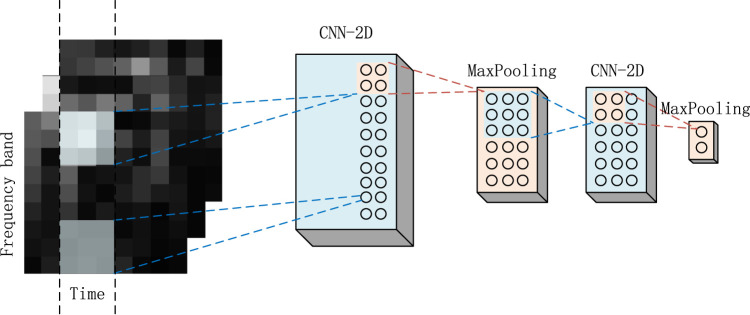
Two-dimensional CNN architecture.

Among them, $b_{l}$ is bias, σ is a nonlinear activation function. A two-dimensional pooling layer is added after each two-dimensional convolution layer to reduce the data dimension and reduce the amount of calculation. Figure 12 is the two-dimensional CNN structure diagram designed in this study.

#### 2.3.7 RNN architecture design

RNN is also a deep learning model. Its core idea is to learn features through cyclic structure, and gradually learn the features and dependencies of input sequences through cyclic processing. Among them, the GRU is a popular variant of RNN. With its gating mechanism, GRU successfully solves the problems of long dependence and gradient explosion or disappearance in traditional RNN.

The one-dimensional CNN architecture designed in this study shows low sensitivity to time sequence when dealing with time series fragments. RNN can effectively capture the temporal features in the data by selectively incorporating and conveying information through hidden state time steps.

**FIGURE 13:**
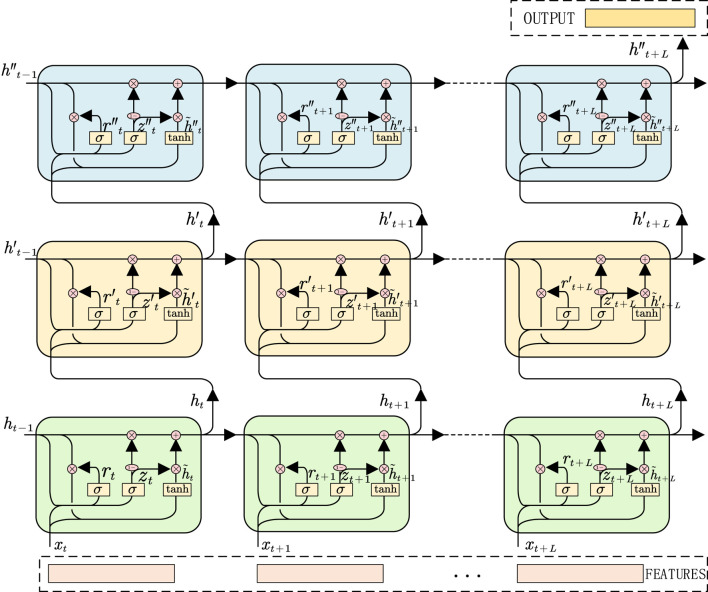
GRU RNN architecture.

Therefore, in order to fully explore the characteristics of time sequence in sEMG, this study introduces a RNN after one-dimensional CNN, and designs a three-layer GRU RNN with one-dimensional CNN to achieve timing sensitivity and lightweight. In addition, for the time-domain characteristics of sEMG, the three-layer GRU RNN is used to further learn the features with the number of features as the time step and the number of channels as the dimension. Figure 13 is the GRU loop network structure diagram designed in this study.

### 2.4 Models’ evaluation

**FIGURE 14:**
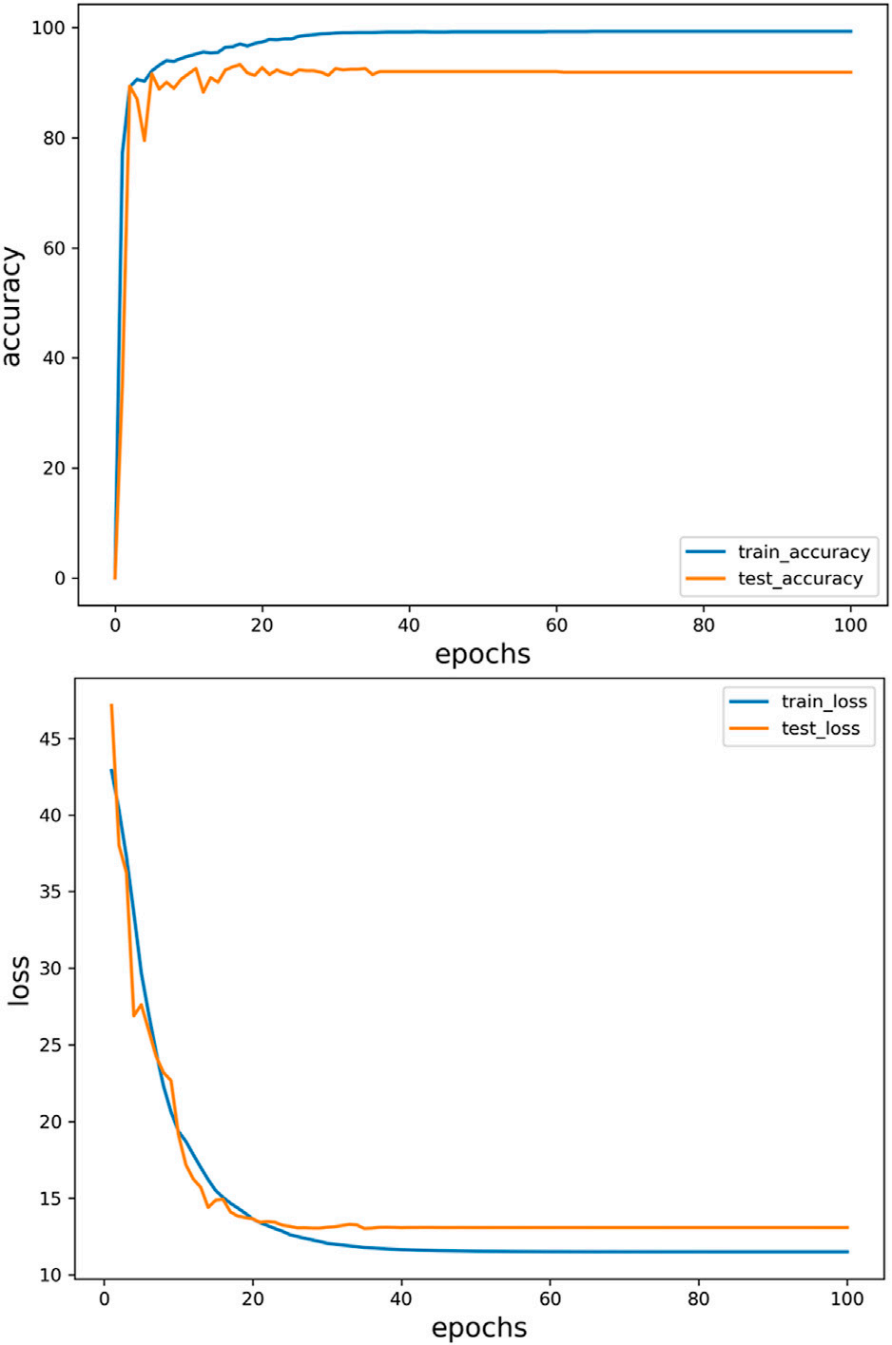
The change curve of gait recognition accuracy and total loss in training process.

In order to evaluate the performance of the designed neural network, this study uses data collected from each volunteer‘s experiment, and divides these data into 80% for model training and 20% for model testing to evaluate the performance of the model. The input is the feature extracted from the surface electromyography signal, and the output is the gait cycle and joint angle. Figure 14 is generated by periodically collecting the output of the model on the verification set during the training period. For the change curve of gait recognition accuracy and total loss of all learning tasks during the training process, the prediction results of the model are recorded at each time point to observe the gradual improvement of gait cycle recognition and joint angle prediction in the learning process of the model. The training achieves better results after about 40 epochs.

This study focuses on the ability of the model to recognize the correct and identify a certain gait. Based on calculating the accuracy and precision of each gait category recognition, the macro average accuracy, micro average accuracy, macro average sensitivity and micro average sensitivity are further calculated. Relevant indicators that can reflect the performance differences of the model in each category recognition are used to comprehensively evaluate the performance of gait recognition. For the prediction of joint angle, two common indicators, root mean square error (RMSE) and cross-correlation coefficient, are used to evaluate the accuracy of joint angle prediction. The RMSE is used to measure the difference between the estimated value and the true value, and the cross-correlation coefficient is used to measure the correlation between the estimated value and the true value.

This chapter describes the method of predicting joint angle based on sEMG signal proposed in this study. Time domain feature extraction and time-frequency domain feature extraction are carried out based on collected signal. The advantages and disadvantages of time domain feature and time domain feature are analyzed. Then, three deep learning neural network architectures are designed based on appropriate feature combinations, including one-dimensional CNN, two-dimensional CNN and GRU RNN. These network architectures can perform effective feature extraction and feature learning on input data through convolution and loop operations for different types of data and task scenarios.

## 3 Results and discussion

### 3.1 Gait recognition

**FIGURE 15:**
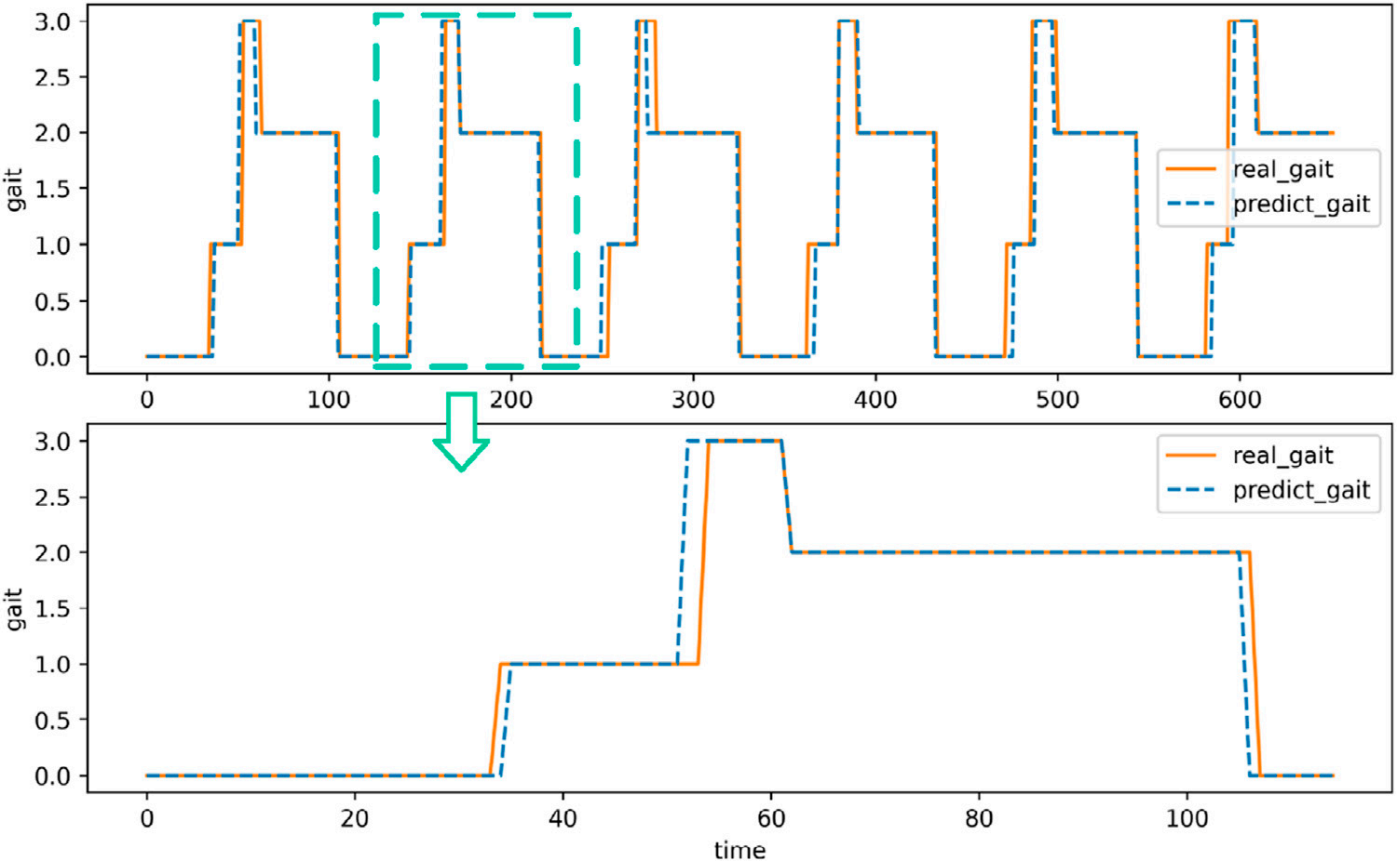
Walking gait recognition of a volunteer on one walk.

The comparison between the continuous gait recognition of a volunteer and the real lower limb walking gait is shown in Figure 15. The results indicate that during the four gait cycles in the test set, the identified gait patterns closely match the actual gait patterns, with most errors occurring in the gait phase transition areas. In the gait transition stage, there will be a certain time error in the recognition of gait compared to the real gait. The primary reason for this time discrepancy lies in the inherent latency of the sensor data processing and the control system’s response time. During real-time gait recognition, there is a slight delay in detecting the transition phases due to the time required to collect and process sensor signals, as well as the computational time for the algorithm to classify the gait phase. Additionally, factors such as noise in the sensor signals and the filtering process to smooth the data can introduce further delays. In the early stage of support, there is also a fluctuation of recognition results. However, in general, in the process of system recognition of walking gait, each stage is in a relatively stable state, and the phase lead or lag is only a very small time period.

The overall situation of the relevant evaluation indicators of all volunteer test data is shown in Table 2. Table 2 indicate that the median of the overall accuracy of the recognition is 92.77%. The recognition accuracy of the swing period (S0) and the end of the support (S2) is slightly higher than that of the initial support (S1) and the middle support (S3). The medians of the identification accuracy in the swing period and the end of the support are 95.24% and 94.39%, respectively, while the medians of the identification accuracy in the early and middle stages of the support are 82.81% and 82.62%, respectively.

**TABLE 2:** Multi-classification performance matrix of walking gait recognition.

index	Category			
	SW(S0)	IC(S1)	TSt (S2)	MSt (S3)
precision/(%)	95.24 ± 3.12	82.81 ± 1.32	94.39 ± 2.98	82.62 ± 1.54
accuracy/(%)	92.77 ± 2.66			
$P_{MAP}$	90.02 ± 1.34			
$P_{MIP}$	95.97 ± 2.67			
$P_{MAS}$	91.40 ± 1.28			
$P_{MIS}$	95.16 ± 2.54			

In practical applications, the initial stage of support and the middle stage of support are one of the main stages of identifying errors. At this time, the errors mainly occur in the swing period and the middle stage of support. From the whole study, this is because the sEMG of these two states have certain similarity characteristics. In the macro-average and micro-average correlation indicators, MAP and MAS are similar, and far lower than MIP and MIS, which indicates that in a cycle walking cycle, the state of small sample data such as the initial and middle support needs more attention.

### 3.2 Joint angle prediction

**FIGURE 16:**
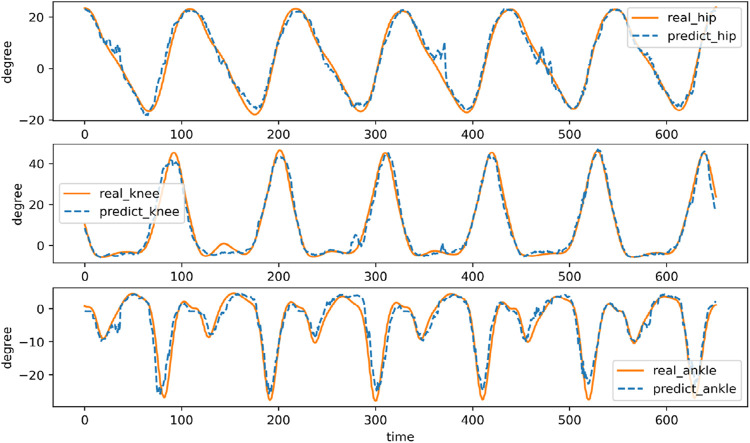
Joint angle estimation of a volunteer walking in one step.

The comparison between the estimated joint angle of a volunteer and the real lower limb joint angle is shown in Figure 16, and the predicted value and the actual value show a very similar trend. In the rising and falling stages of the joint angle curve, the predicted angles of the three joints follow the actual joint angles. In this process, the curve will have a small range of oscillation. At the same time, the predicted curve of the joint angle has a large fluctuation at the maximum and minimum values. On the one hand, there is a large deviation between the predicted value and the actual value, and on the other hand, it is the oscillation at the maximum value. This phenomenon can be attributed to several factors. One key reason is the complexity of predicting joint angles during dynamic transitions, such as the hip flexion-to-extension phase. During this phase, rapid changes in muscle activation and load distribution occur, which can introduce non-linearities that are challenging for the prediction model to capture accurately. Additionally, the model may experience difficulties in accurately representing the biomechanical dynamics of the hip joint, especially during phases of rapid motion, leading to oscillations or deviations in the predicted angle.

The statistical analysis results of the joint angle estimates for all volunteer test data are shown in Table 3. The RMSE of hip joint, knee joint and ankle joint angles are about 2.19,3.51 and 2.57 respectively, and the relative errors are about 5.26%, 4.79% and 7.89% of the three joint angles respectively. The cross-correlation coefficient of the three joints were 0.99,0.99 and 0.96, respectively. The multi-branch deep learning neural network model shows excellent performance, and the recognition and prediction results have lower RMSE and higher cross-correlation coefficient. It is worth noting that the predictive performance of the ankle joint angle is lower than that of other parts, which may be due to the complexity of the ankle joint angle motion curve.

**TABLE 3:** Multi-classification performance matrix of walking gait recognition.

Angle	RMSE/(°)	Cross-correlation coefficient
$\theta_{hip}$	2.19 ± 1.38	0.99
$\theta_{knee}$	3.51 ± 2.14	0.99
$\theta_{ankle}$	2.57 ± 1.22	0.96

### 3.3 Limitations and future work

The human motion intention recognition and joint angle prediction model designed in this study only performs data acquisition and model training on three volunteers, which may lead to under-fitting phenomena and problems in the model. Therefore, it is difficult to establish a neural network model with universality and good generalization ability. In the future research process, more experimental data should be collected for analysis and used for model training to improve the overall performance of the identification and prediction model.

The current human lower limb motion data uses ordinary walking data, but there is a lack of relevant data and analysis for running, jumping, climbing and other sports. At the same time, this study only uses a signal data source of sEMG signal, so the follow-up research work can consider using a variety of sensor information fusion methods to achieve more efficient and reliable human motion recognition of multiple actions.

### 3.4 Strengths and contributions

Combining feature extraction and feature learning, a three-branch deep learning neural network is designed by using the advantages of different neural networks. The time-domain features, time-frequency features and time-series segments of sEMG after signal preprocessing and feature extraction are processed respectively. The recognition and prediction accuracy of human gait and joint angle is high. The core of the lower extremity exoskeleton robot based on sEMG signal control is to obtain the motion intention of the human body through sEMG signal decoding. Traditional machine learning methods perform well on small-scale data sets and are easy to explain and understand. However, they are not effective in dealing with high-dimensional complex data such as sEMG. Feature extraction and selection often require a lot of domain knowledge. Deep learning methods such as CNN and RNN perform well in dealing with high-dimensional data and nonlinear relationships. They can automatically extract features and reduce the difficulty of feature extraction. In addition, different types of deep learning neural networks have their own advantages in dealing with different types of data and task scenarios. Therefore, this study uses this feature to build a multi-branch deep learning neural network, and designs different neural network architectures to process sEMG. Different feature items give full play to the advantages of each branch neural network, thereby improving the accuracy of gait recognition and joint angle estimation, and providing accurate control instructions for lower limb exoskeleton robots.

This chapter evaluates the effect of the multi-branch deep learning neural network. A performance evaluation system is built, including performance indicators such as accuracy, precision, macro-average accuracy, micro-average accuracy, macro-average sensitivity and micro-average sensitivity. These indicators can measure the performance of the model from different angles. By training and testing all the experimental data of all volunteers, it is verified that the multi-branch deep learning neural network has better performance. In addition, this chapter discuss limitations and future work and strengths and contributions.

## 4 Conclusion

In this study, two methods of feature extraction and feature learning are combined. Aiming at the problem of continuous estimation of joint angle by surface electromyography signal. Using the advantages of different neural networks, a three-branch deep learning neural network is designed to process the time-domain features, time-frequency features and time-series fragments of sEMG after signal preprocessing and feature extraction, respectively. The recognition and prediction accuracy of human gait and joint angle is high. The prediction model can be used to design accurate angular biosensors. Then, the prediction model can be applied to all kinds of exoskeleton robots, and the prediction results are used as control instructions to realize more intelligent and more human-machine cooperative intelligent robots. The current human lower limb motion data uses ordinary walking data, but there is a lack of relevant data and analysis for running, jumping, climbing and other sports. At the same time, this study only uses a signal data source of sEMG signal, so the follow-up research work can consider using a variety of sensor information fusion methods to achieve more efficient and reliable human motion recognition of multiple actions.

## Data Availability

The original contributions presented in the study are included in the article/supplementary material, further inquiries can be directed to the corresponding authors.
